# Prevalence and Correlates of Dental Caries in an Elderly Population in Northeast China

**DOI:** 10.1371/journal.pone.0078723

**Published:** 2013-11-19

**Authors:** Lu Liu, Ying Zhang, Wei Wu, Min Cheng, Yan Li, Ruibo Cheng

**Affiliations:** 1 Department of Preventive Dentistry, School of Stomatology, China Medical University, ShenYang, China; 2 Department of Epidemiology, School of Public Health, China Medical University, Shenyang, China; 3 Department of Preventive Dentistry, Stomatological College of Jilin University, Changchun, China; 4 Heilongjiang Provincial oral Disease Prevention Hospital, HaErBin, China; National Taiwan University, Taiwan

## Abstract

**Objectives:**

The present study aimed to investigate the prevalence and correlates of dental caries in elderly population in northeast China.

**Methods:**

A community-based, cross-sectional study among 2376 elderly subjects (age: 65–74 years) from nine urban areas and nine rural areas in three provinces of northeast China was conducted using multistage stratified random sampling per the World Health Organization oral health survey methodology. Decayed-missing-filled teeth (DMFT) and decayed-filled teeth (DFT) indices were used to evaluate the prevalence of dental caries. Face-to-face questionnaire survey on oral health was performed in a randomly selected subset (n = 1197). T test and chi square test were employed to compare groups for continuous and categorical variables, respectively. Multivariate logistic regression was used to estimate odds ratios (ORs) and corresponding confidence intervals (CIs).

**Results:**

67.5% of elderly subjects reported dental caries (average DFT = 2.68±3.40), and the prevalence was higher in urban areas (*P*<0.01). Missing teeth accounted for 80.72% of DMFT, and filled teeth due to caries accounted for 2.08% with a rate higher in urban areas (*P*<0.01). Logistic regression analysis indicated significant association among elderly population in urban areas (OR 1.713; 95% CI 1.337–2.195), smoking (OR 1.779; 95% CI 1.384–2.288), and individuals without dental insurance (OR 2.050; 95% CI 1.120–3.754) with dental caries.

**Conclusions:**

The prevalence of dental caries in the elderly population in northeast China is high. The elderly from urban areas who smoke and who do not have a dental insurance are at a higher risk to develop dental caries.

## Introduction

Aging population is a major challenge for social and economic development and sustainability worldwide [1].This is particularly true in China which has approximately one fifth of the global aged population [2]. Dental caries is a common disease among the elderly, which can result in pain and chewing difficulties, thus decreasing their overall health and quality of life. Epidemiological studies show that the prevalence of dental caries is low among children, adolescents, and middle-aged adults in countries where easy access to health care, preventive measures, and medical insurance system are available [3], [4]. However, dental caries in elderly population, with a prevalence rate from 49.3% to 78.6%, still remain a major concern [5]–[11].

In China, the prevalence of dental caries in the elderly population in different regions ranges from 66.03% to 87.42% [12]–[17]. Recently, the third China National Oral Health Survey (2005) showed that the overall prevalence of dental caries in the elderly Chinese population was 75.2% [18]. This observation emphasizes the importance and need in identifying risk factors and preventive strategies for dental caries among this high risk population. Lin et al suggested that rural residence, female, low educational level, and having no access to regular oral check were the major determinants of dental caries in elderly population in south China [19]. Unfortunately, no comprehensive epidemiological data is available for dental caries prevalence in the elderly population in northeast China. In addition, data regarding the potential risk factors of dental caries in Chinese elderly population, especially in northeast China are not adequate and may vary from those living in south China because of the diversity in climate, economic status, culture, and lifestyle.

The present study, as a part of the third China national oral health survey, represents the largest ever survey conducted in northeast China consisting of three provinces (Liaoning, Jilin, and Heilongjiang), which share similar historical background, geographic characteristics, climate, dialect, and economic status. The populations in these provinces have similar habits and customs and thus can be regarded as a homogenous group. The objective of this study was to investigate the prevalence and correlates of dental caries in elderly population in northeast China.

## Materials and Methods

### Study population

**Figure 1:**
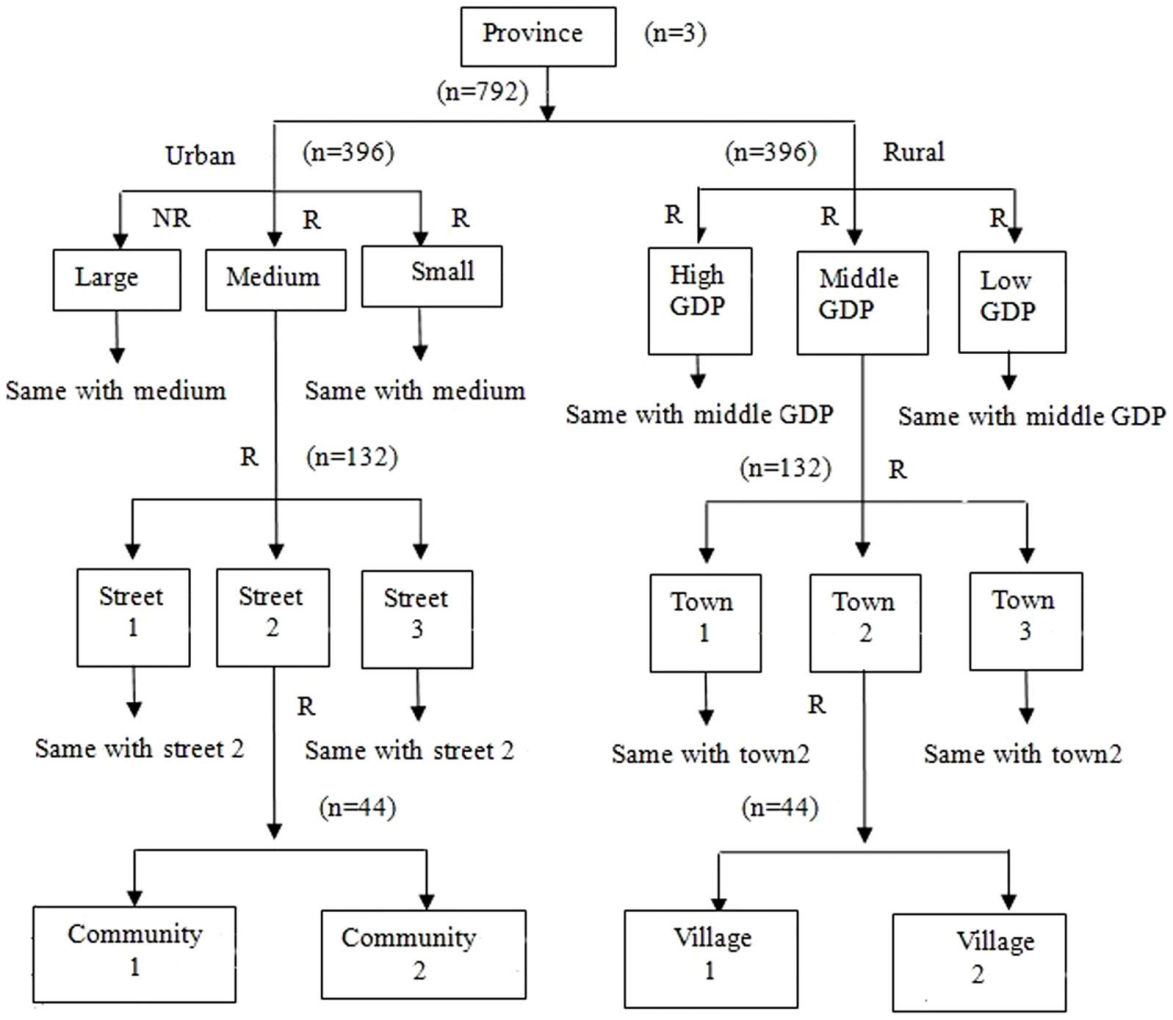
The flow chart shows a multistage stratified random sampling technique of the present study used per the overall design of the third China National Oral Health Survey. The number of subjects participated in the study in each category has been indicated. NR, non-randomized sample; R, randomized sample.

The study protocol was approved by the Ethics Committee of the Stomatology Hospital, China Medical University, Shenyang, China. Written informed consent was obtained from each study participant before enrollment. As per the overall design of the third China National Oral Health Survey [20], a multistage stratified random sampling technique was employed as shown in Figure 1.

In the first stage, urban and rural areas in the three provinces of northeast China were selected. All the urban areas (cities) in each province were divided into three categories (large, medium, and small cities) based on the size of its population. For the purpose of this study, the capital cities of each province (Shenyang for Liaoning province, Changchun for Jilin province, and Harbin for Heilongjiang province) were chosen as large cities. One medium city and one small city were randomly selected from each province. As per this categorization, nine urban areas were selected in total including Shenyang, Jinzhou, and Wafangdian in Liaoning province; Changchun, Siping, and Jiangyuan in Jilin province; and Harbin, Mudanjiang, and Zhaodong in Heilongjiang province. All the rural areas (counties) in each province were also divided into three categories (high, middle, and low) based on its economic status, and one county was randomly selected from each category in each province. The selected rural areas for the study were Liaozhong, Dengta, and Yixian in Liaoning province; Huadian, Meihekou, and Yanji in Jilin Province; and Zhaoyuan, Shuangcheng, and Linkou in Heilongjiang province.

In the second stage, survey units from the selected urban and rural areas were determined. In each district of the selected cities, three streets were randomly selected, and then two communities were randomly selected from each of the selected streets. In each of the selected counties, three towns were randomly selected, and then two villages were randomly selected from the selected towns. From each of the selected community or village, 20 subjects were randomly sampled (male to female ratio, 1∶1). Given the possible decline of survey participants due to invalid questionnaires, two more individuals (10% of the 20 subjects) were selected from each survey unit. Subjects were eligible for the study if they were 65–74 years old and had lived in the sampling sites for more than six months at the time of sampling.

### Quality control

Three examiners and two investigators (all registered dentists) were selected from each province and trained by the third China National Oral Health Survey instructor group as per the World Health Organization (WHO) oral health survey methodology [21]. All the examiners and investigators were requested to pass the standard test before they were certified for the survey (kappa>0.90). During the survey, inter-rater agreement test was also carried out and a high agreement was observed (kappa>0.80). Uniform devices and equipments including dental exam chairs, lights, mouth mirrors, and Community Periodontal Index (CPI) periodontal probes were used in the various sites during the survey.

### Dental examination

Dental examination was carried out for 2376 subjects (sample availability, 99.49%) as per the WHO oral health survey methodology [21]. The number of decayed teeth with crown or root caries (DT), missing teeth due to caries (MT), and filled teeth due to caries (FT) were recorded. DT and FT were summed up to the decayed and filled teeth (DFT) index; and all the DT, MT, and FT were summed up to the decayed, missing, and filled teeth (DMFT) index. The prevalence of dental caries referred to the percentage of subjects who suffered from crown or root caries to the total number of subjects. Crown caries were diagnosed as the presence of either cavities, or destruction under enamel, or soft cave bottom, or lesions of the cave at pits and fissures or smooth surface using the CPI probe. Root caries were diagnosed when the tooth root had been exposed, and soft leather-like lesions were found using the CPI probe. The crown and root caries were recorded separately if they were not observed in the same positions. Single location of caries which involved the crown or root surface was recorded as crown caries or root caries.

### Questionnaire survey

Approximately half the participants were randomly selected to complete the face-to-face questionnaire survey. 1197 valid questionnaires were obtained from the three provinces. General information (residence, gender, and nationality etc.) and information about their oral health knowledge, attitudes, and behaviors; use of fluoride toothpaste; eating habits; and dentist visits were collected. The reliability and validity of the questionnaire were previously tested. The overall Cronbach's α coefficient of all the items in the questionnaire was greater than 0.85, which confirmed the internal consistency reliability. Spearman correlation analyses for all the items and factors showed that the internal consistency coefficient α for each factor was greater than the coefficients between this factor and other factors. And for each item, the correlation coefficient with other items within the factor was greater than those of the other factors. These analyses confirmed the validity of the questionnaire.

### Statistical analysis

Data was entered with EpiData 3.0 (The EpiData Association, Odense, Denmark) and analyzed with SPSS 13.0 (SPSS Inc, Chicago, IL, USA). T-test was used for comparison of continuous variables in two groups. Kolmogorov-Smirnov was applied to test normality prior to the t-test. If the data were not normally distributed, log-transformation was performed. For categorical variables, chi square test was used. Statistical significance was considered if P value was <0.05. To identify possible risk factors for dental caries, univariate analysis (chi square test) was conducted for oral health behaviors, use of fluoride toothpaste, eating habits, and dental visits. The significant variables were included in the multivariate binary logistic regression analysis in which the dental caries status (DFT≥1 versus DFT = 0) was categorized as the dependent variable.

## Results

### Prevalence of dental caries in the study population

Among the 2364 enrolled subjects, 1595 had dental caries (67.5%) and the DFT was 2.68±3.40. As shown in table 1, significant differences in the prevalence of dental caries and DFT index were found between urban and rural areas (for dental caries rate, *P*<0.01; for DFT index, *P*<0.01). No significant difference was observed between male (65.8%) and female (69.2%) (*P* = 0.08).

**Table 1:** Prevalence of dental caries in an elderly population aged 65–74 years old in northeast China.

	Urban areas			Rural areas			Both areas		
	Men	Women	Both	Men	Women	Both	Men	Women	Both
Number of subjects	583	594	1177	591	596	1187	1174	1190	2364
DT									
Mean ± S.D.	2.3±3.28	2.42±3.16	2.36±3.22	2.23±3.46	2.6±3.28	2.42±3.37	2.27±3.37	2.51±3.22	2.39±3.29
Percentage (%)	18.39	16.7	17.47	16.51	16.76	16.92	17.73	16.73	17.19
MT									
Mean ± S.D.	9.78±8.39	11.52±9.55	10.66±9.02	10.76±9.68	12.8±10.57	11.78±10.18	10.28±9.06	12.16±10.08	11.22±9.63
Percentage (%)	78.18	79.5	78.9	82.26	82.59	82.38	80.31	81.07	80.72
FT									
Mean ± S.D.	0.42±0.98	0.55±1.27	0.49±1.14**	0.09±0.37	0.11±0.47	0.10±0.42	0.25±0.76	0.33±0.98	0.29±0.88
Percentage (%)	3.4	3.79	3.6	0.69	0.71	0.70	1.95	2.20	2.08
DMFT									
Mean ± S.D.	12.51±8.84	14.49±9.40	13.51±9.17	13.08±9.79	15.51±10.22	14.30±10.07	12.80±9.32	15.00±9.82	13.90±9.64
DFT									
Mean ± S.D.	2.72±3.42	2.97±3.40	2.85±3.41*	2.32±3.47	2.71±3.29	2.52±3.38	2.52±3.44	2.84±3.34	2.68±3.40
Prevalence (%)	69.5	72.6	71.0*	62.1	65.8	63.9	65.8	69.2	67.5

* P<0.05 and

** P<0.01, compared with rural areas.

Abbreviations: DT, decayed tooth; MT, missing tooth; FT, filled tooth; DMFT, decayed, missing, and filled tooth; DFT, decayed and filled tooth; S.D., standard deviation.

In the DMFT count, missing teeth accounted for the majority (80.72%); and filling teeth due to caries accounted for 2.08%, which was higher in urban areas than that of rural areas (*P*<0.01).

### Correlates of dental caries in the study population

In the univariate analysis, residence (*P*<0.01), smoking (*P*<0.001), and availability of insurance (*P*<0.05) differed significantly between the dental caries cases and controls; and thus, they were included as independent variables in the logistic regression analysis (Table 2).

**Table 2:** Univariate analysis of potential risk factors in relation to prevalence of dental caries in an elderly population aged 65–74 years in northeast China.

Factors	Categories	No. of participants	No. of cases with dental caries	*x* ^2^	P
Areas	Urban	598	434		
	Rural	599	386	9.178	0.002**
Gender	Men	593	400		
	Women	604	420	0.602	0.438
Smoking frequency	Every day or every week	424	256		
	Seldom, never, or ceased	773	564	20.100	7.349×10^−6^ **
Tooth brushing frequency	Once or more per day	729	485		
	Six times or less per week	468	335	3.371	0.066
Toothpaste types	Fluoride	344	246		
	Other types or no toothpaste	853	574	2.023	0.155
Seeing a dentist for decayed teeth	Yes	312	217		
	No	885	603	0.214	0.643
Having insurance or reimbursement	Yes	49	26		
	No	1148	792	5.510	0.019*
Last time to see a dentist	One year ago or earlier	989	677		
	Less than one year	208	143	0.007	0.933

* P<0.05;

** P<0.01.

In the multivariate logistic regression analysis, urban residence was inversely associated with the odds of dental caries (OR = 1.713; 95% CI = 1.337–2.195), while smoking (OR = 1.779; 95% CI = 1.384–2.288) and having no dental insurance or reimbursement (OR = 2.050; 95% CI = 1.120–3.754) were significantly and positively associated with the odds of dental caries (Table 3).

**Table 3:** Multivariate logistic regression analysis of potential risk factors in relation to prevalence of dental caries in an elderly population aged 65–74 years in northeast China.

Variables	β	S.E.	OR	95% CI	P
Urban area	0.538	0.127	1.713	1.337–2.195	2.105×10−5**
Smoking	0.576	0.128	1.779	1.384–2.288	6.964×10^−6^ **
Having no insurance or reimbursement	0.718	0.309	2.05	1.120–3.754	0.020*

* P<0.05;

** P<0.01.

Abbreviations: β, regression coefficient; S.E., standard error; OR, odds ratio; CI, confidence interval.

## Discussion

In this survey, we found that the prevalence of dental caries was 67.5% in elderly population in northeast China, and the DFT index was 2.68±3.40. The observed prevalence is close to those observed in several previous studies conducted in Beijing (66.03%; 2.37±2.96) [15], India (64.2%) [22], and Hungary (2.04±1.45) [23]; but higher than those reported in Canada (49.3%; 1.62±2.53) [6] and that of the second China National Oral Health Survey in 1995 (64.8%; 2.41±3.10) [24]. This indicated that dental caries is still a common and frequently-occurring disease, which did not improve alongside economic development. As a large geographic region in China, northeast part of China was one of the most advanced industrial bases of northeast Asia in the 1930s. However, the economic development in northeast China lagged behind south China, because of the reform and economic policies in those coastal regions. The decline in economic growth in northeast China in turn slowed down the improvement of the people's lifestyle, civilization, health awareness, and behaviors, which might have affected the behaviors of oral health among the elderly in northeast China and led to the increasing prevalence of dental caries. In addition, the elderly population might have more difficulties in getting access to dental care service compared with other age groups. There could be several factors beyond economic status contributing to it, including geographical proximity of the dental clinic, difficulty in travelling (such as mobility, the need for assistance, fatigue, and medical conditions), and some management barriers in specialized institutions. Jointly, these factors could hinder the elderly population to attain dental care services and thus lead to a higher risk of developing dental caries. This poses a challenge to the local oral health resources and calls on the social engagement to raise attention to oral health in the elderly population. Oral health education and health promotion oriented to the elderly population should be involved as one of the public health priorities in the future [25].

The present survey showed that prevalence of dental caries in the elderly of northeast China was higher in urban areas than that in the rural areas (P<0.01), which was similar to the results from other studies conducted in Asian (66.1% vs 61.4%) and African regions (39.2% vs 34.6%) [22], [26]. This could be as a result of longer life span and lower rate of missing teeth (78.90% vs 82.38%) among the elderly who live in urban areas than those who live in rural areas. Longer retention time of teeth will lead to a higher rate of dental caries [22]. There was also evidence suggesting that in some regions of China, the differences in prevalence of dental caries between urban and rural areas are narrowing along with the socioeconomic development. Furthermore, the prevalence of dental caries in some rural areas was even higher than that in the urban areas [27], [28]. Studies have shown that oral health morbidity is related to the socioeconomic status of an individual/population [29]. In an elderly population, oral health variables, income, diet and social class are correlated. Social variables such as marital status and lifestyle factors such as alcohol abuse are also shown to be related to oral health of the elderly [30]. As a result, the narrowing differences between urban and rural areas may be related to the socioeconomic development in rural areas recently, which led to changes in diet and lifestyle in some elderly in rural areas.

Prevalence of MT in the elderly in northeast China accounted for 80.72% of DMFT, which was higher than the national average level (75.3%) [18] and similar to that of the study from Brazil (79.01%) [26]. Only 2.08% of the elderly received filling treatment, while the majority of dental caries remained untreated. Significant difference of filling treatment between urban and rural areas was found (*P*<0.01), which was similar to Shah's conclusion [22]. This indicated that dental caries were largely untreated in the elderly in the northeast China, especially in rural areas. In developed countries, FT accounted for the majority of the DMFT [19], and this could be related with the improved welfare system and health awareness in those countries. In China, the elderly are more prone to compromised oral health because of low income, limited access to dental care services, and limited healthcare knowledge. Most of the elderly individuals in the northeast China, especially in rural areas prefer to remove a seriously decayed teeth rather than having it restored, leading to more MT and less FT. Although there is a potential growth of oral health institutions and relevant resources in recent years, these resources are not appropriately distributed in urban and rural areas [25]. In addition, the dental insurance system in northeast China is still not satisfactory, and majority of the residents have to pay for oral health care. Therefore, it is a priority to better distribute oral health resources in this region and to strengthen effective utilization of those resources including oral health service.

Among all the potential factors, urban residence, smoking, and having no dental insurance or reimbursement were significantly associated with the prevalence of dental caries in the elderly northeast China. The elderly living in urban areas had greater odds of suffering from dental caries, which was similar to findings in the previous studies (P<0.01) [18], [22], [26]. The high socioeconomic lifestyles make people more susceptible to dental caries [22], [31]. China is one of the industrialized countries, and many heavy industries are located in urban areas in the northeast, especially since in the 1930s. The main labor force in urban areas at that time is now becoming elderly population. Long-term exposure due to work in heavy pollution environment increased the chance of suffering from chronic diseases [32], [33]. Many elderly subjects in urban areas consume multiple medications for chronic systemic conditions like cardio-respiratory problems, high blood pressure, and psychiatric illness, which may cause xerostomia, increasing the dental caries risk, when compared to the rural elderly who had worked at farms [34], [35]. This may also be the reason why the findings from the elderly population of southern China [19] differ from the present study results.

Annual tobacco sales in China account for 30% of those around the world [36]. Among the elderly in northeast China, 35.4% smoke, which was higher than the national average level (26.9%); and smoking percentage in the elderly female in northeast China was 22.7%, which was much higher than the national average level (8.8%) [18]. Smoking can increase the risk of calculus; debris accumulation; gingival recession; and attachment of bacteria plaque to the cemento-enamel junction and root surface, leading to the changes of cementum structure, demineralization, and occurrence of dental caries [37]. Poor oral health caused by smoking is one of the risk factors for dental caries in the elderly in northeast China, which was unique comparing with other parts of China.

Having no dental insurance or reimbursement is also a risk factor for dental caries in the present study population. In this study, we found that the prevalence of dental caries among the elderly in northeast China who had dental insurance or reimbursement (comprising 4.09% of all the elderly in this region) was 53.06%, while the prevalence in those who did not have was 68.99%. Besides the limited coverage of dental insurance in this area, this may be also a result of poor self-health awareness [19]. Most elderly people did not recognize their dental caries as a disease, or they thought their dental caries did not need any dental care. In our questionnaire survey, only 13.97% of the elderly regularly visited a dentist for oral health checkup or had preventive treatments. Most of them only sought dental treatment only because of acute toothache, trauma, or other problems. Dental floss was used in only 0.47% of the population, and most of them never used dental floss. Based on the questionnaire, only 26.07% of the subjects chose to seek dental care, when they realized that they had holes in teeth.

Fluoride toothpaste, sugar-sweetened foods and beverages, and teeth brushing frequently were also found to be related to dental caries in previous studies [9]. These factors were also investigated in this study, but no statistical significance correlation was observed. In the present study population, 39% of the elderly brushed their teeth less than once a day, 28.67% used fluoride toothpaste, and the number was even smaller (12.33%) for those who consumed sugar-sweetened foods and beverages on a daily basis.

In this study, both the prevalence of dental caries and DMFT were analyzed, and both of them can reflect the severity of dental caries. The results facilitate direct comparison among different population groups [21]. However, there are still several limitations in the present study. First, only DMFT/DFT cases were recorded without including the decay of, missing, or filled tooth surface. It was not required to check the tooth surface caries as per the study design of the third China national oral health survey, although gingival recession was recorded [20]. Secondly, as per the WHO guideline for adults over the age of 45 years old [21], both MT due to caries and MT due to periodontal diseases were coded as missing rather than separately reported. Thus MT was not counted in analyzing the prevalence and mean. Also, in the logistic regression analysis on correlates of dental caries, the DFT was used as the dependent variable. The prevalence of MT was high in elderly population in northeast China, and the leading cause was smoking-induced periodontal diseases. If MT was included in the statistical analysis, MT due to periodontal disease might have led to substantial bias. Third, some potential factors that contribute to dental caries were not surveyed in this study such as salivary flow rate, use of dentures, and consumption of dairy products which had been previously shown to contribute the incidence of dental caries [5], [38]. The questionnaire used in this study was issued by the Chinese Ministry of Health, and the factors mentioned above were not covered and needs to be evaluated in future studies.

In summary, findings from this study indicate high prevalence of dental caries and low filling rate in the elderly population in northeast China. The elderly who live in urban areas, smoke, and without dental insurance are at a higher risk to develop dental caries. The study indicates that oral health education should be strengthened in this region and dental insurance should be made available. Government agency in northeast China may need to take measures to establish and improve the primary oral health care system, which may help promote the elderly population's healthy lifestyles, meet oral health needs, and reduce the incidence of dental caries.
